# Emerging Biomarker Potential of Extracellular Vesicle-Enclosed MicroRNAs for Liver Fibrosis Detection

**DOI:** 10.3390/cells14131025

**Published:** 2025-07-04

**Authors:** Sharmila Fagoonee, Valeria Menchise, Daniela Delli Castelli, Stefania Bruno

**Affiliations:** 1 Institute of Biostructure and Bioimaging (CNR), Molecular Biotechnology Center “Guido Tarone”, 10126 Turin, Italy; valeria.menchise@unito.it; 2 Department of Molecular Biotechnology and Health Sciences, Molecular Biotechnology Center “Guido Tarone”, University of Turin, 10126 Turin, Italy; daniela.dellicastelli@unito.it; 3 Department of Medical Sciences, University of Torino, 10126 Turin, Italy

**Keywords:** fibrosis, chronic liver diseases, extracellular vesicles, microRNAs, biomarkers

## Abstract

Liver fibrosis is a frequent pathological outcome of long-term liver diseases, arising from sustained damage to the liver. Two main types of liver damage can trigger fibrotic progression: hepatocellular injury, often caused by viral infections, alcohol, or metabolic disorders, and cholestatic injury, associated with impaired bile flow due to autoimmune or congenital conditions. Despite diverse etiologies, liver fibrosis exhibits conserved biological processes, including hepatocyte death, chronic inflammation, disruption of epithelial or endothelial barriers, and excessive deposition of extracellular matrix (ECM) components. These coordinated events reflect the complex interplay among parenchymal damage, immune activation, and fibrogenic signaling pathways. If unresolved, fibrosis may progress to cirrhosis, liver failure, or hepatocellular carcinoma. In the pursuit of non-invasive biomarkers for early detection and monitoring of fibrosis, extracellular vesicles (EVs) have garnered significant attention. Among the diverse cargoes within EVs, microRNAs (miRNAs) have emerged as particularly promising due to their stability, disease-specific expression patterns, and involvement in fibrogenic signaling. This review explores the role of EV-associated miRNAs in liver fibrosis, highlighting key candidates implicated in hepatocellular and cholestatic injury and their clinical potential as diagnostic and prognostic biomarkers, with special focus on MAFLD/MASH, primary sclerosing cholangitis, primary biliary cholangitis, and biliary atresia as representatives.

## 1. Introduction

Liver fibrosis represents a common pathological outcome of various chronic liver diseases and develops as a consequence of sustained hepatic damage. It is characterized by aberrant deposition of extracellular matrix (ECM) components, primarily type I and type III collagens, which eventually form fibrous tissue that distorts liver architecture and compromises function. Different hepatic cell types orchestrate collagen deposition, including activated hepatic stellate cells (HSCs), liver sinusoidal endothelial cells (LSECs), and portal fibroblasts [1]. Liver fibrosis can be broadly classified into two categories: hepatocellular and cholestatic injuries [2]. Hepatocellular injury results from direct cellular damage due to external agents such as chronic viral infections (hepatitis B and C), excessive alcohol intake leading to alcoholic steatohepatitis (ASH), or from metabolic disturbances causing non-alcoholic steatohepatitis (NASH), as well as from chronic immune-inflammatory responses such as autoimmune hepatitis [3,4]. On the other hand, cholestatic injury is caused by impaired bile secretion or flow, often linked to disorders such as primary sclerosing cholangitis (PSC), primary biliary cholangitis (PBC), or congenital anomalies like biliary atresia [4]. These diseases are liver conditions involving damage to the bile ducts, marked by ongoing inflammation, impaired bile flow, and biliary fibrosis [5]. As these diseases advance, they can lead to cirrhosis, increased pressure in the portal vein, and eventual liver failure. Despite the multiple origins of liver injury, fibrosis progression follows a set of conserved mechanisms, including hepatocyte loss, persistent inflammatory signaling, breakdown of tissue barriers such as the sinusoidal endothelium or biliary epithelium, and ECM deposition [6]. These events reflect the complexity of hepatic fibrogenesis, which integrates a dynamic interplay between parenchymal injury, immune responses, and fibrogenic signaling pathways. If left unchecked, fibrosis can progress to cirrhosis, liver failure, or hepatocellular carcinoma (HCC). Therefore, early detection, staging, and monitoring of fibrosis are critical for disease management and therapeutic intervention.

The current gold standard for assessing liver fibrosis is liver biopsy, despite the drawbacks of being invasive, subject to sampling errors, and unsuitable for serial monitoring [7]. In the race to search for biomarkers in recent years, extracellular vesicles (EVs), lipid bilayer-bound particles released by cells and found in all body fluids, have emerged as a promising non-invasive source of biomolecules for early detection of liver fibrosis [8,9,10]. EVs enclose microRNAs (miRNAs), which are small non-coding RNAs (typically 18–24 nt long) that post-transcriptionally regulate gene expression and participate in homeostasis maintenance or disease promotion [11]. These EV-derived miRNAs hold particular promise due to their stability, disease specificity, and involvement in fibrogenic pathways. In this review, we examine EV-encapsulated miRNAs implicated in liver fibrogenesis and highlight their potential as diagnostic and prognostic biomarkers in both hepatocellular and cholestatic liver injury.

## 2. Extracellular Vesicle Isolation and Purification for Biomarker Search

EVs encompass a heterogeneous population of vesicles, mainly categorized as exosomes (30–150 nm) generated via the endosomal pathway, microvesicles (100–1000 nm) shed directly from the plasma membrane, and apoptotic bodies (>1000 nm) formed during programmed cell death. Surface markers on extracellular vesicles, including exosomes, microvesicles, and apoptotic bodies, reflect their cellular origin and biogenesis pathways, offering valuable insight for distinguishing between vesicle subtypes and enabling their use in diagnostic and therapeutic applications. For instance, specific surface markers such as CD81, CD63, Alix, and Tsg101 on exosomes, Caspase 3 and histones on apoptotic bodies, and selectins, integrin, and CD40 on microvesicles can help differentiate EV subtypes [12,13,14]. Due to their pivotal role in mediating intercellular communication from donor to recipient cells, EVs are increasingly recognized as versatile agents in biomedical applications. These nanosized vesicles, secreted by nearly all cell types, carry a plethora of proteins, lipids, and nucleic acids that provide a molecular snapshot of their cells of origin. For example, tumor-derived exosomes have been shown to transport oncogenic biomolecules that can influence the tumor microenvironment and promote metastasis [15]. This cargo-loading capacity positions EVs as promising vehicles for targeted drug delivery, allowing therapeutic molecules to be transported directly to specific tissues or cell types with high precision and reduced systemic toxicity [16,17]. Furthermore, because the molecular contents of EVs can mirror disease-specific changes, they are being actively explored as non-invasive biomarkers for conditions such as cancer, neurodegenerative disorders, liver failure, and cardiovascular disease [18,19,20,21]. These features collectively highlight the potential of EVs not only as diagnostic tools but also as platforms for novel therapeutic strategies.

### 2.1. Isolation and Purification of Extracellular Vesicles for Clinical Biomarker Applications

The clinical potential of EVs as biomarkers relies heavily on the ability to isolate and purify them effectively from complex biological fluids (biofluids) such as blood, urine, and cerebrospinal fluid. However, EV isolation presents substantial technical challenges due to their small size, heterogeneity, and the presence of contaminating proteins and lipoproteins in biofluids [22]. The efficiency of retrieving EVs from plasma varies based on the chosen isolation technique. Several methods have been developed, each with unique strengths and limitations, especially concerning yield, purity, scalability, and compatibility with downstream molecular analyses.

#### 2.1.1. Differential Ultracentrifugation

Differential ultracentrifugation is one of the most widely used traditional methods for EV isolation from cell culture media or from body fluids such as serum. It involves sequential centrifugation steps at increasing speeds to remove cells, debris, and finally to pellet EVs [23]. With this procedure, EVs do not require particular pretreatment prior to their isolation. However, although effective, this method is time-consuming, labor-intensive, and often results in co-purification of protein aggregates or lipoproteins, hence reducing EV purity. The latter is an important consideration in clinical diagnostics [24].

#### 2.1.2. Density Gradient Centrifugation

To improve purity and yield, density gradient centrifugation (e.g., sucrose gradients) can be employed after ultracentrifugation. This approach separates EVs based on their buoyant density, helping to discriminate between EV subtypes and contaminants [25]. However, density gradient centrifugation remains a low-throughput technique and is not easily automated, limiting its applicability in high-volume clinical settings. Moreover, EV functionality may be impaired due to the excessive force applied during ultracentrifugation. Contemporary precipitation-based approaches have emerged as substitutes for ultracentrifugation, offering advantages such as minimal input volume requirements from human biofluids and compatibility with high-throughput workflows. However, isolating EVs from plasma remains particularly challenging due to the inherent viscosity and high protein content of plasma, which adversely affect the purity achievable through ultracentrifugation techniques [26]. More recently, a streamlined method utilizing a single-step size-exclusion chromatography (SEC, described below) process has been introduced for isolating EVs directly from human plasma.

#### 2.1.3. Size-Exclusion Chromatography (SEC)

SEC separates EVs from other particles based on hydrodynamic properties and size. It is a gentle, reproducible, and scalable method that preserves EV integrity and is especially compatible with clinical applications [27]. Lobb et al. compared different isolation methods for retrieving EVs from plasma and demonstrated that ultrafiltration coupled with SEC had superior efficiency in terms of purity compared to other precipitation methods, such as ultracentrifugation or charge-based precipitation, for isolating EVs from human plasma. This was confirmed recently in another study showing the reproducibility, simplicity, and purity of SEC-based separation of EVs in the clinical setting from 1 mL of plasma, and its suitability in the clinical setting [28]. Despite its utility, SEC may result in the co-elution of particles with comparable dimensions, like protein clusters or lipoproteins, and typically necessitates additional concentration procedures.

#### 2.1.4. Polymer-Based Precipitation

Water-excluding polymer-based precipitation methods (e.g., using polyethylene glycol and PEG) offer a simple, rapid, and scalable solution to isolate EVs from various fluids [29]. Exosome precipitation kits also provide higher EV yields of miRNAs and mRNAs for high-throughput molecular analyses [30]. These kits are widely used in clinical research due to ease of use [31]. However, they typically yield low-purity preparations with significant contamination from soluble proteins, which can hinder tissue-derived biomarker discovery [32].

#### 2.1.5. Immunoaffinity Capture

Immunoaffinity-based methods use antibodies against specific EV surface markers (e.g., CD63, CD9, and CD81) to selectively capture vesicles of interest. For instance, the use of anti-CD81 V_H_H ligand-based immunoaffinity chromatography methods allows for the direct capture of EVs from cell culture supernatant, showing strong potential in enriching disease-specific EV populations with high specificity [33,34]. However, the high cost, limited scalability, and dependence on well-defined surface markers restrict its general application.

#### 2.1.6. Microfluidic and Lab-on-a-Chip Technologies

Advancements in microfluidic platforms, commonly known as lab-on-a-chip systems, have paved the way for streamlined EV capture, refinement, and characterization in compact, efficient formats suitable for clinical applications [35]. These microscale devices replicate conventional isolation methods by precisely directing fluid behavior within confined channels. Among their key advantages are rapid processing times, reduced consumption of biological material and reagents, multiplexing capabilities, and compatibility with automated high-throughput protocols. By integrating diverse separation mechanisms such as antibody-based targeting, electric field-driven sorting, and membrane-based sieving, these tools enable high-purity EV isolation from limited sample inputs. Despite their potential, many of these technologies remain in the developmental phase or are undergoing preliminary clinical validation.

Identifying the most effective method for isolating EVs remains a major hurdle in their clinical application. When evaluating these approaches, the ideal protocol would be straightforward, cost-effective, avoid reliance on complex or high-end instrumentation, deliver results quickly, and be capable of processing large sample volumes efficiently [36]. The effectiveness of any EV isolation technique hinges on how well it performs with human clinical specimens, especially when EVs are being investigated as potential new biomarkers. All these procedures, applied individually, produce different yields of EVs with different grades of purity. Integrating multiple enrichment techniques can improve the purity of EVs, thereby strengthening the accuracy and validity of subsequent experimental analyses, which may help in finding the right combination for a cost-effective procedure for the clinical setting. For instance, merging PEG-based precipitation with ultrafiltration can provide highly pure small EVs nearly void of non-vesicular particles or artifacts [37].

### 2.2. EV Quality Testing

Quality testing of EVs is crucial, since EVs are highly heterogeneous, and their quality can significantly affect the reliability of results. Quality testing involves a multifaceted approach that combines physical, biochemical, and functional assessments to ensure the suitability of EV preparations for different applications. The MISEV guidelines were recently updated and can help researchers in standardizing the different techniques used to characterize EV preparations in order to obtain comparable results among different laboratories [38]. Dynamic light scattering and nanoparticle tracking analyses are commonly used to determine EV size and concentration. Transmission electron microscopy is used to assess the integrity of EV membranes. Assessing zeta potential, a measure of the surface charge of EVs, can confirm the integrity of EVs. EVs are negatively charged in biofluids and zeta potential effects EV stability, aggregation, and interaction with target cells. Specific proteins, such as tetraspanins (CD63, CD9, and CD81), can serve as markers to identify EVs, and different techniques, such as ELISA, flow cytometry, and western blotting, are commonly used to detect these markers.

### 2.3. EV Composition

EVs carry nucleic acids, proteins and lipids, which can influence the behavior of recipient cells and hold potential as diagnostic biomarkers or as vehicles for drug delivery [14]. Among nucleic acids, RNA is the most abundant and is predominantly short in size (less than 200 nucleotides (nt) long) [39,40]. Sequencing of total RNA from serum-derived EVs showed that miRNAs and transfer RNA constitute around 15% of all RNA species contained inside these nanoparticles [41]. The presence of long transcripts (more than 200 nt long) as well as coding and non-coding RNAs have also been reported in EVs [40,41]. Interestingly, some RNAs, especially miRNAs were enriched in the released EVs, in comparison with the cells of origin [42,43,44]. Circular RNAs (circRNAs) are also abundant in circulating EVs. RNA-seq analyses indicated that they are enriched in EVs compared to the cells of origin; more than 1000 circRNAs were identified in human serum exosomes [45]. EVs can transport DNA ranging in size from 100 base pairs (bp) to 2.5 kilobase pairs (kB) [46,47,48]. EV-DNA reflects parental cell genomic DNA, as cancer-related mutations in genes such as BRAF, EGFR, KRAS, and TP53 have been identified in DNA from EVs derived from melanoma and pancreatic cancer cells [48,49].

The protein composition of EVs is influenced by both the cell of origin and the mechanism of vesicle formation. Exosomes originate from the endosomal compartment and are enriched in major histocompatibility complex class II (MHC class II) and tetraspanins, and in the endosomal sorting complex required for transport (ESCRT) that constitutes a group of proteins indispensable for the formation of multivesicular bodies [50,51,52]. The ESCRT pathway requires accessory proteins, such as Alix and tumor susceptibility gene protein 101 [52]. Owing to their plasma membrane origin, MVs are enriched in a different set of proteins, such as integrins, glycoprotein Ib, and P-selectin [50]. EV proteins can be grouped in functional classes. For example, nucleic acid binding is the most common function among proteins present in EVs, together with catalytic activity [53,54]. Moreover, in EVs, transcription factors, receptors, enzymes, and functional metalloproteases that can induce phenotypic changes in recipient cells can be found [55].

The lipid bilayer of EVs resembles that of the plasma membrane. Exosomes are enriched in gangliosides, sphingomyelin, and desaturated lipids, and phosphatidylcholine and diacylglycerol proportions are lower than in their cells of origin [56]. An increased presence of cholesterol in exosomes compared with the membranes of cells of origin has been reported [57].

During EV biogenesis and secretion, factors such as membrane composition, surface proteins, and cellular origin shape the biomolecular corona, the dynamic layer of proteins and other biomolecules that adsorb onto the EV surface [58]. Once released, EVs encounter diverse extracellular environments that can alter or reshape this corona, influencing their stability, uptake, and functionality. The biomolecular corona of EVs constitutes a dynamic and functionally significant layer that plays a central role in mediating EV interactions with recipient cells. During EV biogenesis within the cell, a subset of proteins associates with the vesicle surface, forming a nascent corona. Following secretion, this corona evolves as additional proteins and biomolecules from the extracellular environment bind to the EV surface, giving rise to what is commonly referred to as the protein corona [58]. Emerging evidence indicates that the EV corona is not limited to proteins alone but also encompasses other classes of biomolecules, including lipids, RNA, and even DNA. This expanded composition forms what is increasingly recognized as the EV biomolecular corona. Pathological conditions are often accompanied by changes in cellular physiology, which are reflected in the composition of secreted EVs, both in their internal cargo and surface-associated molecules. These disease-specific alterations in the EV biomolecular corona present a valuable opportunity for biomarker discovery. Given its accessibility and responsiveness to pathophysiological states, the EV biomolecular corona holds significant promise as a diagnostic tool, particularly for the early detection of diseases [59].

## 3. Intercellular Communication via EVs in Liver Fibrosis

**Figure 1:**
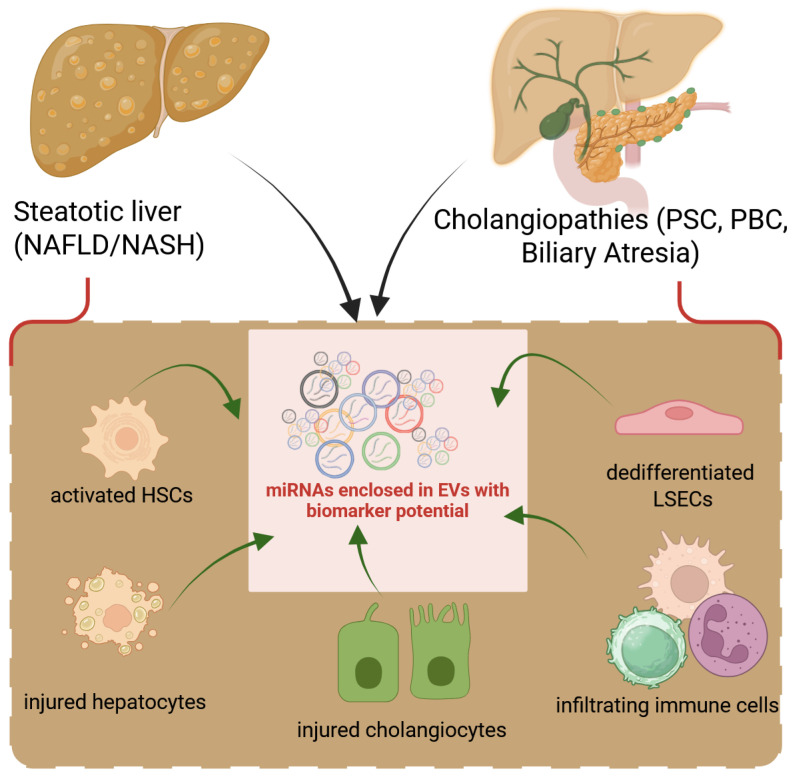
Liver cells release EVs to perpetuate fibrotic signals. Following damage to hepatocytes (hepatocellular) or cholangiocytes (biliary), all hepatic cell types react by releasing growth factors, cytokines, or EVs into the secretome, which lead to the dedifferentiation of LSECs followed by activation of HSCs or fibroblasts and dysregulated production of ECM. Infiltrating inflammatory cells also contribute to liver fibrosis with their load of secreted factors.

Persistent hepatocyte damage leads to inflammation, activation of fibrogenic pathways, and excessive ECM deposition, which gradually distorts liver architecture and impairs function. Liver fibrosis arises from a complex interplay among various cellular and molecular components, beginning with hepatic injury and progressing to irreversible ECM deposition. Signaling between hepatocytes, HSCs, Kupffer cells, LSECs, and immune cells plays a central role in the initiation and perpetuation of fibrogenesis (Figure 1).

The liver, as a central metabolic organ, constantly communicates with other tissues via secreted molecules, including EVs. The resident liver cells’ secretome, which is its repertoire of bioactive factors, exerts a profound influence on liver fibrogenesis (Table 1).

Input from mice models and clinical samples has demonstrated the utility of EVs as a biomarker source in the context of liver fibrosis. EVs secreted from any cell type of the injured liver can participate in damage perpetuation. Thus, identifying EVs carrying loads of fibrotic indicators could facilitate early diagnosis and serve as a valuable diagnostic marker. The EV contents vary according to disease etiology, as shown in Table 2.

Recent studies have reported that specific subpopulations of EVs are released by organelles, for instance by mitochondria, and are enriched with mitochondrial components, which can influence the phenotype and metabolic state of recipient cells [70,71]. The mitochondrial content of EVs appears to vary under pathological conditions. For instance, in a murine model fed an ethanol-enriched diet, plasma levels of hepatocyte-derived EVs containing mitochondrial components were elevated and this was suggested to contribute to liver injury by promoting inflammation and endoplasmic reticulum stress [72]. Similarly, EVs isolated from in vitro ethanol-treated human or murine hepatocytes exhibited increased levels of mitochondrial RNA. These EVs induced IL-1β expression in Kupffer cells, thereby promoting inflammation through the activation of a specific T lymphocyte subpopulation [73]. Collectively, this evidence suggests that alterations in the quantity or composition of circulating EVs enriched in mitochondrial components could serve as potential biomarkers for pathological conditions, particularly in alcoholic liver disease.

### 3.1. Biofluids with Biomarker Utility for Liver Fibrosis

Biofluids, such as serum, bile, and urine, are rich in biomarkers for detecting liver fibrosis and these fall into several categories: proteins, metabolites, nucleic acids, and lipids, as well as EVs. The latter reflect the molecular state of the cells of origin and are detectable in several biofluids, notably serum, bile, and urine. Each fluid offers unique insights into liver pathophysiology, disease biomarkers, and therapeutic monitoring.

#### 3.1.1. Blood

Serum and plasma are the most commonly used biofluids for assessing liver fibrosis due to their accessibility and the abundance of potential biomarkers. Alanine aminotransferase (ALT) and aspartate aminotransferase (AST) are routinely measured enzymes that indicate liver injury, although they do not correlate specifically with the stage of fibrosis. Elevated levels of gamma-glutamyl transferase (GGT) and alkaline phosphatase (ALP) are more suggestive of cholestasis and advanced fibrotic changes [74,75]. Components of ECM can also be detected in serum and serve as direct indicators of fibrogenesis [76]. For example, hyaluronic acid (HA), a marker of ECM remodeling, increases in circulation with the progression of fibrosis. Similarly, procollagen III N-terminal peptide (PIIINP), a precursor of collagen type III, correlates well with fibrosis severity in infantile cholestasis [77]. Another notable marker is cytokeratin-18 (CK-18), which reflects hepatocyte apoptosis and is particularly elevated in metabolic dysfunction-associated steatohepatitis (MASH)-related fibrosis [78]. In addition to proteins, serum contains circulating non–EV-bound microRNAs, such as miR-122 and miR-34a. In an effort to distinguish between non-hepatitis B- and non-hepatitis C-related HCC and NAFLD patients or healthy controls, Boonkaew et al. analysed plasma EV miRNA profiles. The results pointed to five EV miRNAs comprising miR-19-3p, miR-16-5p, miR-223-3p, miR-30d-5p, and miR-451a as elevated in the HCC group compared to controls, with EV miR-19-3p being revealed as the best diagnostic predictor of Alfa-fetoprotein-negative and early HCC [79]. On the other hand, EV miR-223-3p was statistically significantly higher in NAFLD patients with respect to controls, suggesting its potential as a biomarker for this pathology.

#### 3.1.2. Bile

Bile is a complex fluid secreted by the liver, and is a viable and promising source of biomarkers for liver and biliary tract cancers because of its proximity to the disease site [80]. Human bile contains inorganic electrolytes, lipids (bile acids, phospholipids, and cholesterol), bilirubin, and proteins, and is abundant in EVs that can be isolated and characterized using standard protocols, typically yielding vesicles of ~30–200 nm in size [81,82].Bile-derived EVs have been reported to contain higher amounts of Claudin 3 protein in Cholangiocarcinoma (CCA) cohorts compared to biliary stone disease patients, showing potential as a diagnostic marker for CCA [83]. Moreover, bile offers a rich, disease-relevant EV miRNA milieu that surpasses serum in biomarker concentration and specificity, making EV-derived miRNAs particularly useful for detecting cholestatic and biliary tract diseases. For instance, significantly higher levels of bile exosomal miR-200 family members (including miR-200a-3p and miR-200c-3p) have been found in patients with CCA compared to those with benign diseases, such as biliary stone disease [84]. Moreover, pairing the bile EV miRNAs miR-200a-3p/-200c-3p with serum CA19-9 greatly enhanced diagnostic performance. Bile EV analysis on larger human cohorts during ERCP has identified miR-451a and miR-3619-3p as robust biomolecules for distinguishing biliary tract cancers from non-malignant controls [85]. Notably, higher expression of miR-3619-3p was also linked to a worse prognosis in patients with biliary tract cancers. Technically, accurate quantification of bile EV miRNAs requires rigorous normalization procedures, like the inclusion of synthetic spike-in controls such as Cel-miR-39, in order to mitigate variability introduced during RNA extraction [86].

#### 3.1.3. Urine

Urine is a valuable, accessible and non-invasive biofluid, which is rich in EVs. Urinary EVs can be purified by methods such as SEC or precipitation, and they contain stable, vesicle-encapsulated miRNAs derived from the urogenital tract and also from various tissues. Although urinary EV miRNA levels may be lower than those in serum due to renal filtration, disease-specific signatures have been detected in liver pathology contexts. Lipidomic profiling of urinary EVs from NAFLD and NASH patients revealed four lipid molecules (free fatty acid (18:0), lysophosphatidylcholine (22:6/0:0), free fatty acid (18:1), and phosphatidylinositol (16:0/18:1), capable of high discrimination of NALFD cohorts that progress to NASH [87]. Moreover, in CCA, specific EV transcripts reflecting tumor tissue were detected in urine, despite the fact that the overall number of potential biomarkers in urine may be lower than in serum, a result of renal filtration processes [88]. In non-viral hepatocellular carcinoma (HCC), plasma EV miR-19-3p showed strong diagnostic accuracy, showing excellent detection capability for early-stage and AFP-negative HCC [79].

Due to their accessibility in the clinic, these biofluids are regarded as ideal sources of EVs for biomarker discovery and analysis. Differences exist between EVs derived from biofluids and those isolated directly from tissues. In fact, growing evidence indicates that tissue-derived EVs contain a richer repertoire of biological information and more precisely reflect changes in the tissue microenvironment compared to EVs derived from cultured cells or biofluids [89]. Tissue-derived EVs are usually found at significantly lower concentrations than EVs derived from circulating blood components. Consequently, molecular analysis of tissue-derived EVs in biofluids necessitates stringent pre-analytical handling and highly reproducible analytical methodologies to ensure accurate and reliable data acquisition (https://www.biomedcentral.com/collections/EVPCLIP; accessed on 1 July 2025). The origin of tissue-derived EVs is more well-defined, allowing for more precise tracking using electron microscopy. However, their isolation typically requires tissue explants or biopsies, which are inherently invasive procedures. For example, to isolate liver-derived EVs, whole livers were harvested from carbon tetrachloride-treated mice [90]. Therefore, at present, EVs derived from biofluids remain the most practical and minimally invasive option for biomarker discovery in disease diagnostics.

## 4. EV-Derived miRNAs as Biomarkers for Liver Fibrosis

### 4.1. NAFLD/NASH

Non-alcoholic fatty liver disease (NAFLD), recently renamed metabolic dysfunction-associated steatotic liver disease (MASLD), is the most common chronic liver disease in Western countries and affects about a quarter of the world’s adult population [91,92]. MASLD encompasses several pathological conditions, from steatosis to liver inflammation and fibrosis (non-alcoholic steatohepatitis (NASH), now known as metabolic dysfunction-associated steatohepatitis (MASH)), which can progress to cirrhosis and carcinoma [91]. Recently published studies have investigated the use of EVs as a non-invasive tool for NAFLD/MASLD and NASH/MASH diagnosis and staging.

Higher levels of circulating EVs were found in the plasma of mice and humans with chronic liver diseases compared to healthy controls [93,94,95]. The correlation between circulating EV levels and disease severity has been demonstrated in various mouse models of NAFLD and NASH [96]. For example, the number of circulating EVs increased with the progression of NAFLD and correlated with liver fibrosis in diet-induced NAFLD in mice. Circulating EVs in the NASH mouse model were enriched with miR-122 and miR-192, two miRNAs typically expressed in hepatocytes [97,98]. In the sera of NAFLD patients, EV–associated miR-122 and miR-192 were described as candidate biomarkers of NASH progression [99,100]. In addition, the expression of EV-derived miR135a-3p was found to be lower in the serum of patients with NAFLD compared to healthy controls, and ROC analysis suggested that this EV-derived miRNA may be a sensitive biological marker for the diagnosis of NAFLD [101]. A recent publication reported that miR-122 and miR-128 were upregulated, while miR-200, miR-298, and miR-342 were downregulated in EVs isolated by ultracentrifugation from plasma samples of patients with NAFLD and NASH compared to normal controls [102]. Similarly, EVs isolated from serum using a commercially available isolation kit contained specific miRNAs that correlated with inflammation, steatosis, ballooning, and NASH scores [103].

The selective isolation of circulating EVs derived from hepatocytes represents an opportunity to improve the use of EV-derived miRNAs as biomarkers for liver disease. Liver-derived EVs can be isolated by immunoprecipitation with anti-sialoglycoprotein receptor 1. The EVs isolated from NASH patients’ livers showed enrichment of several miRNAs (miR-122, miR192, and miR-128-3p) compared to those obtained from NAFLD patients and healthy controls, suggesting that these EV miRNAs are significantly associated with liver disease severity [104]. This study emphasizes the possibility of isolating liver-specific EVs to identify biomarkers for NAFLD that reliably distinguish patients with NAFLD and NASH.

The various techniques commonly used for the purification of EVs, such as ultracentrifugation and selective selection by immunoprecipitation, are time-consuming, work-intensive, and costly, which limits their applicability in the clinical setting. An innovative approach of capturing EVs from serum using wheat germ agglutinin-coupled magnetic beads and real-time quantitative PCR to analyze EV miRNAs, showed that miR-574-3p, miR-542-3p, and miR-200a were significantly elevated in patients with MASLD [105]. These miRNAs are involved in various processes associated with the development of liver fibrosis and chronic liver disease. For example, miR-574-5p was found to be upregulated in serum EVs and positively correlated with collagen deposition and alpha-smooth muscle actin (α-SMA) expression in liver tissue during fibrosis [106]. Furthermore, miR-542-3p and 200a-3p were significantly upregulated in liver fibrosis and promoted the activation of HSCs [107,108]. These results suggest that EV miR-574-3p, miR-542-3p, and miR-200a-3p may play a role in the occurrence and progression of MASLD and could be used as diagnostic markers.

### 4.2. Cholestatic Injury-Related Liver Fibrosis

**Figure 2:**
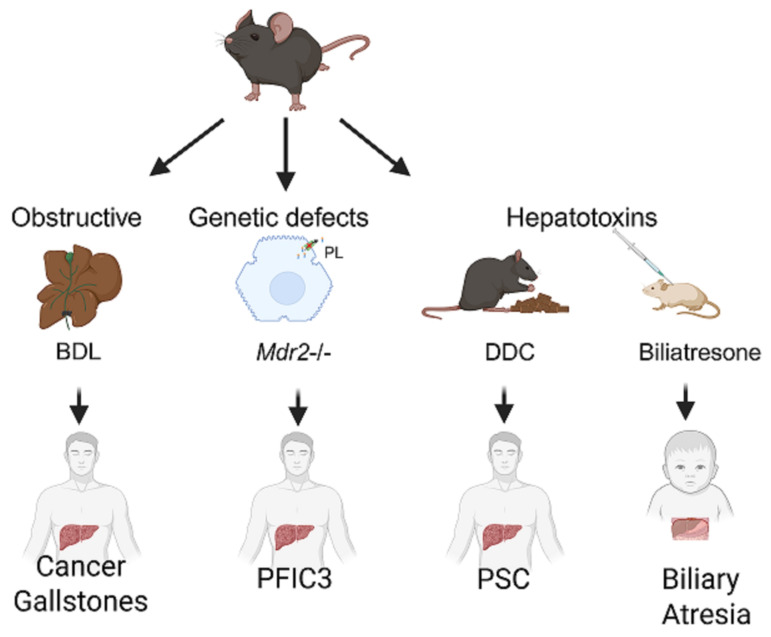
Mouse models of human biliary tract diseases. Representative models of biliary damage caused by bile duct obstruction, genetic defects, or exposure to hepatotoxins are shown, with clinical translatability regarding cancer and gallstones, progressive familial intrahepatic cholestasis type 3 (PFIC3), primary sclerosing cholangitis (PSC) and biliary atresia, respectively, in humans. BDL—bile duct ligation; Mdr2—multidrug resistance 2 (Abcb4); DDC—3,5-diethoxycarbonyl-1,4 dihydrocollidine.

Very few studies have been performed on the potential use of EV miRNAs as biomarkers for human cholangiopathies. Valuable insights regarding the potential of EV miRNAs as biomarkers for biliary tract diseases have come from preclinical studies. Several mouse models for cholestatic injuries have been developed for biomarker discovery for human biliary tract diseases (Figure 2).

For instance, by inducing biliary injury in mice through obstruction (bile duct ligation (BDL)) or diet (3,5-diethoxycarbonyl-1,4-dihydrocollidine, DDC), and by using genetic models (*Mdr2*-/- mice), we have shown that circulating EV miRNAs such as miR-122-5p, miR-192-5p, and miR-29a-3p closely mirror disease progression following different causes of biliary injury, and can distinguish stages of liver fibrosis [8].

#### 4.2.1. PSC

PSC, a chronic cholestatic disorder with genetic and environmental etiologies, affects bile ducts both at the intrahepatic and extrahepatic levels. EV miRNAs are emerging as promising non-invasive biomarkers for cholangiopathies, but only few data are available for PSC, a chronic cholestatic liver disease with limited diagnostic and prognostic tools. Specific EV miRNAs have shown altered expression profiles in PSC patients compared to healthy controls and those with other liver diseases. Notably, miR-122-5p and miR-192-5p, both liver-enriched miRNAs, were consistently and significantly upregulated in EVs isolated from PSC patients versus healthy controls, reflecting hepatocyte or cholangiocyte injury and serving as markers of disease activity [109]. Additionally, miR-4645-3p, recently identified through miRNA sequencing, exhibits approximately fourfold higher expression in PSC-derived EVs, with its levels correlating closely with miR-122-5p and miR-192-5p, thereby enhancing diagnostic specificity [109]. These EV miRNA signatures not only distinguish PSC from healthy states but also show potential to differentiate PSC from other cholestatic disorders such as PBC or overlap syndromes. More clinical studies should be undertaken to increase the repertoire of EV miRNAs to increase their potential for early diagnosis, risk stratification, and monitoring therapeutic response in PSC.

#### 4.2.2. PBC

PBC is a chronic disorder, characterized by bile retention in the liver. Although its exact cause is unknown, the condition is believed to result from immune-mediated damage to intrahepatic bile ducts. Without treatment, the disease can advance to liver scarring, cirrhosis, and eventually liver failure, highlighting the importance of early diagnosis. Tomiyama et al. investigated the role of plasma-derived EV (exosomes) miRNAs in PBC and identified several upregulated and downregulated miRNAs, with miR-451a and miR-642a-3p being the most significantly upregulated in patients compared to healthy controls [110]. These miRNAs were shown to modulate the expression of co-stimulatory molecules, specifically CD86 and CD80, on antigen-presenting cells such as monocytes in vitro. The regulatory effect suggested a potential link between circulating EV miRNAs and immune dysregulation in PBC. These findings highlight the possibility that EV-associated miR-451a and miR-642a-3p are not only reflective of disease activity but may also contribute to its pathogenesis, underscoring their promise as non-invasive biomarkers for early detection or monitoring of disease progression.

#### 4.2.3. Biliary Atresia

In biliary atresia, the intra- and extra-hepatic bile ducts are progressively destroyed in infants, making this disease the most common cause of pediatric cholangiopathy-associated liver transplantation. Early diagnosis is essential for an appropriate treatment. Albeit EV-associated miRNAs have shown promise as early, non-invasive biomarkers for cholangiopathies, to our knowledge, not much work has been performed regarding biliary atresia. Studies have been restricted to non-EV-enclosed miRNA profiling in serum. Notably, employing a microfluidic array platform, elevated levels of miR-200a, miR-200b, and miR-429 have been observed in BA patients when compared to individuals with other types of neonatal hyperbilirubinemia (validated in *n* = 24 infants, in each group) [111]. Another study, using miRNA expression profiling by microarray analysis, revealed that miR-4429 was downregulated and miR-4689 was upregulated in serum, both showing potential utility in distinguishing BA (validated in *n* = 45 biliary atresia infants and *n* = 30 non-BA neonatal cholestatic controls) [112].

## 5. Clinical Utility of EV-Derived miRNAs in Liver Fibrosis

As described above, the evaluation of circulating EV miRNA contents has emerged as a promising area for discovering new biomarkers in chronic liver disease onset and progression. Development of EV miRNA-based biosensors and liquid biopsy platforms could facilitate non-invasive, real-time assessment of liver health. Panels of EV miRNAs (for instance, miRNA-122-5p and miRNA-409-3p,) may improve sensitivity and specificity when combined with serum markers (e.g., ALT and AST) or scores like aspartate aminotransferase to platelet ratio (APRI) and Fibrosis-4 (Fib-4) [113]. Elevated levels of certain EV miRNAs (e.g., miR-199a-5p and miR-21) are associated with worse clinical outcomes and may predict fibrosis progression or risk of cirrhosis, indicating the utility of EV miRNAs as prognostic indicators. EV miRNAs can also help in response to therapy monitoring, especially in this era of new anti-fibrotic drug design and testing. Changes in circulating EV miRNA levels can reflect treatment response to antifibrotic drugs or lifestyle interventions in MAFLD/MASH and viral hepatitis.

Although a growing body of evidence indicates the feasibility of adopting EV miRNAs as markers of specific liver diseases or for the staging of pathological conditions, there are currently no ongoing clinical trials for EV miRNA large-scale applications as screening or monitoring for liver disease progression. Encouraging results have been recently published showing a comprehensive analysis of plasma EV-associated miRNAs in patients with alpha-1 antitrypsin deficiency (AATD) with and without liver disease, in comparison with non-AATD controls [114]. The objective was to identify miRNAs that could offer insights into liver disease status associated with AATD and help in recognizing individuals with AATD who are at risk of developing hepatic complications. AATD is a rare inherited disorder caused by a point mutation in the SERPINA1 gene, leading to misfolding and polymerization of alpha-1 antitrypsin (AAT) within hepatocytes, which can contribute to liver pathology. Moreover, the low level of AAT in plasma patients induces lung inflammation. Oshins et al. identified six circulating EV miRNAs (let-7a-5p, let-7f-5p, miR-15b-5p, miR-223-3p, miR-23a-3p, and miR-374a-3p) as potential molecular signatures for the detection of AATD liver disease, offering a valuable tool for early detection and risk stratification. The use of circulating EV miRNAs as non-invasive liver disease signatures is advantageous for both clinicians and patients. Indeed, the EV miRNA panel permits detection of liver disease in AATD patients early to start therapeutic interventions before cirrhosis and HCC development. Moreover, the EV miRNA signature could replace the need for liver biopsies that are invasive and risky for patients. Notably, the identified EV miRNA signature may also be useful to monitor responses to anti-fibrotic therapeutic treatments. The use of circulating EV miRNA signatures opens a new perspective in precision medicine to predict severity and progression of disease and to monitor responses to therapeutic interventions.

## 6. Technical Challenges and Considerations

The absence of standardized protocols for EV isolation can result in variability and inconsistency across studies. Selecting a method that offers high purity and yield, while remaining cost-effective and feasible in resource-limited research or clinical settings, remains a key challenge. Accurate quantification of EV-associated miRNAs requires highly sensitive and reproducible techniques, such as digital PCR or next-generation sequencing. However, consensus on robust normalization strategies is still lacking, complicating data interpretation. The expression levels of EV miRNAs can also be significantly influenced by individual factors including comorbid conditions, medication use, and age, introducing further variability in biomarker studies. In systemic circulation, distinguishing EVs derived from specific liver cell types remains technically challenging, limiting the ability to assign functional relevance or diagnostic specificity to EV miRNA signatures. In disease contexts such as MASH/MAFLD, PSC, and biliary atresia, this variability adds complexity to EV-based biomarker discovery, as EV composition and surface signatures may differ depending on both disease state and microenvironmental factors.

## 7. Conclusions and Future Directions

EV-associated miRNAs demonstrate significant potential as non-invasive biomarkers for the detection, staging, and monitoring of liver fibrosis. Their expression levels correlate with fibrogenic pathways, and they are readily detected in accessible body fluids, such as blood, for instance in the case of steatotic liver diseases, while research is still lagging behind in cases of cholangiopathies-associated fibrogenesis. EV corona-associated biomolecules could potentially develop into disease-associated biomarkers and require further investigation. Regarding the clinical application of EV-based biomarkers, the greatest breakthrough in the clinical setting will come from the possibility to enrich EVs from drop-sized blood samples. Blood volume sufficiency EV analysis remains challenging. Recently, Guerrero-Alba et al. employed the PEG-based NTI-EXO method to successfully isolate EVs from just two drops of blood [115]. Several challenges remain, including the standardization and harmonization of isolation, detection, and normalization methods across laboratories to enable reproducible EV miRNA measurements. Large multicenter validation trials in patient cohorts are also needed to confirm the performance of EV miRNA biomarkers and to develop tailored EV miRNA signatures for distinct fibrotic etiologies, such as MASH or cholestatic liver diseases. Gradually, EV miRNA-based biomarker panels are making their way into clinical practice through coordinated research efforts worldwide. However, the world of EVs is under constant evolution as tools for analyzing these nanoparticles are increasing in resolution and throughput, and we are gathering multidisciplinary knowledge about their potential. One of the latest studies shows another tremendous discovery in the EV field. Blebbisomes, unusually large, functional extracellular vesicles that are actively released by human and mouse cells, are capable of moving independently and can both internalize other EVs and release exosomes and microvesicles [116]. How this may impact the biomarker profile across liver diseases remains to be explored.

## Figures and Tables

**Table 1:** Endogenous liver cell secretomes influences on fibrogenesis (including insights from preclinical models). Some examples of biomolecules released by hepatic cell types are shown. NO—nitric oxide; CCL2—C-C motif chemokine ligand 2; TNF-α—tumor necrosis factor-alpha; TGF-β—transforming growth factor-beta; PDGF—platelet-derived growth factor; DAMP: damage-associated molecular pattern; MASP1—mannan-binding lectin serine protease 1; OPN—osteopontin; TGF-α—transforming growth factor-α; FGF—fibroblast growth factor. ↑ and ↓ represent increased or decreased, respectively.

Cell Type	Secretome Factors Released	Consequence on Fibrogenesis	References
LSECs	↓ NO production and restored fenestrae, ↑ TNF-α/chemokines, TGF-β, PDGF, angiocrine EVs	Loss of HSC quiescence; promotion of HSC activation, migration, and ECM deposition; capillarization precedes fibrosis; NO maintains HSC quiescence	[60,61]
Hepatocytes	Injured hepatocyte EVs enriched in DAMPs, miR-27a, and miR-181a; β-arrestin 1 enhances the release of MASP1-enriched EVs	Direct activation of HSC (↑ α-SMA, collagen, and TIMP-1) and immune cell stimulation	[62]
HSCs	PDGF, endothelin-1, CCR2/5, IL-6, leptin, activin, OPN, TGF-α, and FGF	Incites proliferative, angiogenic, or proinflammatory effects on hepatocytes and endothelial cells; attracts inflammatory cells	[63]
Kupffer cells/macrophages	TNF-α, TGF-β, PDGF, and CCL-2	Promotes inflammation and HSC activation	[64]

**Table 2:** EVs released by the different hepatic cell types and examples of their roles in fibrogenesis.

Component	Role in Fibrosis	EV Involvement	Reference
Hepatocytes	Initiate injury via oxidative stress, DAMPs, and cytokines	EVs carry lipotoxic molecules (e.g., ceramides) and pro-fibrotic miRNAs	[62,65]
Macrophages	Promote inflammation through cytokines (e.g., IL-1β and TNF-α)	EVs enriched in inflammatory mediators and microRNAs	[66]
LSECs	Undergo capillarization and lose quiescence signals to HSCs	EVs reflect endothelial stress; modulate vascular inflammation and HSC activation	[1,67]
HSCs	Transform into myofibroblasts; secrete ECM (collagen I/III)	EVs perpetuate activation loops; stimulate release of pro-inflammatory cytokines from immune cells; contain fibrogenic proteins and RNAs	[68]
Fibrocytes	Modulate ECM deposition; may transdifferentiate into myofibroblasts	Pro-angiogenic activity; induce increase in the expression of collagen α1(I) [Colα1(I)] and α-SMA	[69]

## Data Availability

Not applicable.
